# Safe Elective Surgical Practice During COVID-19 Pandemic – A Prospective Observational Study of 303 Elective Surgeries in the UK

**DOI:** 10.7759/cureus.16984

**Published:** 2021-08-07

**Authors:** Chiranjit De, Saumil Shah, Kusy Suleiman, Zehong Chen, Vishal Paringe, Divya Prakash

**Affiliations:** 1 Trauma & Orthopaedics, Sandwell & West Birmingham NHS Trust, Birmingham, GBR

**Keywords:** covid-19, elective surgeries, safety, outcome, protocols

## Abstract

Aim

During the COVID-19 pandemic, there has been worldwide cancellation of elective surgeries to protect patients from nosocomial viral transmission and peri-operative complications. With the unfolding situation, there is a definite need for an exit strategy to reinstate elective services. Therefore, more literature evidence supporting exit plans for elective surgical services is imperative to adopt a safe working principle. This study aims to provide evidence for safe elective surgical practice during the pandemic.

Methodology

This single centre, prospective, observational study included adult patients who were admitted and underwent elective surgical procedures in the trust’s COVID-free environment at the Birmingham Treatment Centre between May 19 and July 14, 2020. Data were collected on demographic parameters, peri-operative variables, surgical specialities, COVID-19 reverse transcription polymerase chain reaction (RT-PCR) testing results, post-operative complications and mortality. The study also highlighted the protocols it followed for the elective services during the pandemic.

Results

A total of 303 patients were included with mean age of 49.9 years (SD 16.5) comprising of 59% (178) female and 41% (125) male. They were classified according to the American Society of Anaesthesiologist Grade, different surgical specialities and types of anaesthesia used. Ninety-six percent (96%) of patients were discharged on the same day. Hundred percent (100%) compliance with pre-operative COVID-19 RT-PCR testing was maintained. There was no 30-day mortality or major respiratory complications.

Conclusion

Careful patient selection, simultaneous involvement of the pre-assessment and anaesthetic team, strict adherence to peri-operative protocols and delivering vigilant post-operative care for COVID-19 infection can help provide safe elective surgical services if the community transmission is under reasonable control. However, it is particularly important to maintain COVID-free safe environment for such procedures.

## Introduction

World Health Organization declared COVID-19 infection as a pandemic on March 11, 2020 and soon it outspread to different continents quickly [1]. Therefore, it was of particular concern for most of health care providers to be prepared to encounter a high number of critically sick patients [2-5]. Therefore, different guidelines were proposed reinforcing the widespread cancellation of elective surgeries worldwide [6-11].

Patients undergoing surgery are particularly susceptible to COVID-19 exposure in the hospital environment and could be vulnerable to further post-operative complications owing to immunosuppressive responses following surgery and pro-inflammatory cytokine surge [12,13]. Therefore, the aim of these extreme measures of cancelling elective surgeries was crucial to protect the patients from nosocomial transmission and associated complications from the disease. At the same time, it intended to protect the health care workers as well. There has been an extensive re-structuring of the human workforce during this critical hour resulting in the redeployment of theatre staff to more demanding specialities. Thus, it led to a high number of pending elective surgeries. The COVIDSurg Collaborative Group estimated the total number of adult elective operations cancelled worldwide could be 28,404,603 during the 12-week peak (2,367,050 per week), accounting for over 2,000,000 cases per week [14]. This clearly reflects the rolling-over pressure on the NHS. To cope with this situation, the National Health Service (NHS) England has adopted safe measures to resume elective services in a safe COVID-free environment and our hospital enacted those terms in between the pandemic waves.

Globally as of June 27, 2020, there have been 9,653,048 confirmed cases of COVID-19, including 491,128 deaths, reported by the World Health Organization [14]. The United Kingdom had been severely affected with 43,514 confirmed cases by then, has the greatest death toll in Europe and is third in the world after the United States and Brazil [15]. We mentioned this timeline as it is roughly the middle of our study period. Due to the high number of cancelled surgeries globally, a high number of pending elective procedures has been continuously increasing the pressure on the already compromised health care systems [16].

We have reported on a prospective study involving adult elective surgeries performed in a COVID-19-free hospital environment during the COVID-19 pandemic. This study aims to show that meticulous patient selection involving adequate pre-assessment and strict compliance to peri-operative protocols, can be a safe way to resume elective surgeries in a designated COVID-19-free environment.

## Materials and methods

This prospective study included patients who underwent elective surgical procedures in the trust’s COVID-free environment at Birmingham Treatment Centre between May 19 and July 14, 2020. This observational study collected single centre data without any change to the set clinical practice. We only included adult patients, excluding all non-elective cases. The project was registered with the trust’s Clinical Effectiveness Department and was conducted as per the local clinical governance protocols. We maintained full compliance to the standards of strengthening the reporting of observational studies in epidemiology (STROBE) statement [17].

The British Orthopaedic Association outlined the concept of ‘Green Pathway’ in their initial guidelines on the resumption of elective services [18]. Theoretically, it might be possible to define a COVID-19-free hospital. However, in a practical scenario, it would be challenging to keep one such. By meaning, ‘Green Corridors’, we would like to refer to a COVID-19-free healthcare environment that includes a “COVID-19-free building” where there are no proven or suspected COVID-19-infected patients. Although it is designed to be a day-surgery unit, overnight in-patients’ capacity is also present in this environment. There should be sufficient space to follow social distancing guidelines between the patients. In our COVID-19-free hospital, patients were kept in a single occupancy room or cubicle without any accompanying persons. Furthermore, all health care professionals working in this hospital building were dedicated to elective services exclusively to minimise the nosocomial transmission of COVID-19 infection compared to the health care professionals who had been working at COVID-19-hot sites. Only the operating surgeons and anaesthetists who were also working in other departments could have been potentially exposed to the virus.

As per the locally implemented protocol, all the healthcare personnel used specific scrubs while at the ‘Green Corridors’ and those scrubs were disinfected after each session. There was absolutely restricted use of these uniforms beyond the clinical corridors. Adequate and multiple handwashing, sanitization and use of shower facilities were encouraged to reduce nosocomial transmission. Furthermore, symptomatic screening of the health care personnel was reinforced. For any prodromal symptoms and suspicion, staff were tested as per NHS testing protocols and self-isolation was advised as per government guidelines [19]. Staff only returned to the ‘Green Corridors’ once they were cleared by occupational health norms considering self-isolation or reverse transcription-polymerase chain reaction (RT-PCR) testing or both. No formal asymptomatic staff testing was undertaken at the time of the study.

A total of 303 consecutive adult patients aged 18 and over who underwent an elective surgical procedure in the COVID-free environment were included in the study. This study includes patients from various specialities including orthopaedic surgery, urology, general surgery, plastic surgery, gynaecology, and ENT (ear, nose and throat) surgery. The patients were meticulously selected through a well-designed pathway. Surgical teams from respective specialities worked synchronously with the anaesthetic and pre-assessment team to screen through the long waiting list to select safe and suitable patients in whom benefit from surgery would be more than the potential risk. All cases were double-checked by one of the designated senior speciality members who finalised the operating lists. Cases were also categorised as per the urgency and complexity. Length of hospital stay following these surgeries was also considered while finalising these lists with priority for day cases. These cases were anticipated for the least complications. Therefore, the anaesthetic and pre-assessment team screened those lists. Stratification of risk factors was also carried out keeping in mind diabetic status, age more than 70 years, presence of ischaemic heart disease, significant chronic lung disease and BAME (Black, Asian and Minority Ethnic) risk assessments. Special attention was given whether patients were on steroids or immunosuppressants and suffering from any condition lowering immunity.

The listed patients were either contacted or reviewed by the operating team to establish the suitability for the surgery and to explain the procedure once again. They were also re-consented explaining all the associated risks once again. Patients were screened for any recent clinical features of a COVID-19 infection like fever, cough, dyspnoea, myalgia, anosmia, or other respiratory symptoms [20]. Moreover, particular attention was given to any possible contact with a COVID-19-infected person in the immediate pre-operative period.

As national guidance was introduced, the pre-operative assessment included a clear directive for self-isolation for at least two weeks before and two weeks after the surgery. As a prospective model of the study, we have been obliged to adhere to all the latest guidance from the Royal College of Surgeons England [6]. Since April 29, 2020, all patients planned for an elective procedure must have had a negative RT-PCR test within 72 hours from the day of the scheduled surgery [21]. It was imperative that the COVID-19 swab result must be available before admission on the day of the surgery. All patients were consented using a separate form adequately mentioning the potential risk for acquiring COVID-19 infection during hospitalization and its possible consequences.

Demographic parameter of these patients was noted with the American Society of Anaesthesiologist (ASA) Grade classification. We also documented different types of anaesthetic procedures noted during the surgical procedure.

Apart from routine post-operative follow-up, they were contacted over the telephone with a validated questionnaire after two weeks of the surgical procedure. The primary outcome of the study was to assess the 30-day mortality rate. We also measured secondary outcomes considering post-operative infection rate and incidence of swab positive results for COVID-19, the necessity for hospital re-admission in the high dependency units/intensive care units and incidence of COVID-19-related respiratory complications. Prospectively, we also checked the electronic medical records (Cerner; Unity) from our hospital database and GP (General Practitioner) records to track if there were any complications, hospital reattendances, or GP visits.

Operating protocols

Standard operating protocols for elective surgery were followed with an additional checklist for COVID-19 swab result (ensuring negative result), a new consent form explaining COVID-19-related complications and also checking for any symptoms of COVID-19. No changes were adopted to the laminar airflow system and sterilization protocols. PPE checks and extra cleanliness were taken care of.

We took measures to minimize the healthcare-related exposure to all the staff members during the peri-operative procedures. Particular attention was given towards in-theatre intubation and extubation, limiting staff members during surgical and aerosol-generating procedures, ensuring sufficient air changes between the procedures; thereby minimizing aerosol contamination and potential transmission. The patients were transferred to the recovery area with an appropriate surgical mask. The recovery area consists of isolated bays or separate cubicles to ensure adequate separation of the patients. Hence, this helped to reduce the chance of nosocomial transmission. The theatre team members ensured that they wore adequate protective equipment (PPEs) during perioperative care. This included protective double gloves, fluid repellent gowns, eye protection and masks according to their FIT testing. Regular FIT testing was carried out for all the staff members to make sure that they are at par with the safety benchmark before allocating them in these ‘Green Corridors’.

## Results

The demographic profile of the study population is summarized in Table 1. During the period of the study, a total of 324 patients were listed for surgery following pre-operative assessment. However, 21 patients were cancelled because they failed to qualify the strict pre-operative protocols considering symptomatic, tested positive for COVID-19, failing to self-isolate, being in contact with someone symptomatic or tested positive. Therefore, in total, 303 patients underwent an elective surgical procedure at the Birmingham Treatment Centre during the study period between May 19 and July 14, 2020. The mean age of the patients was 49.9 with a standard deviation of 16.5 years.

**Table 1:** Demographic parameters for the patients. SD – Standard Deviation, ASA – American Society of Anaesthesiologist

Total Patients (N)	303
Gender	
Male	125 (41%)
Female	178 (59%)
Age (Years)	
Mean	49.94
SD	16.45
Age groups (Years)	
≤45	127 (41.9%)
46 - 64	110 (36.3%)
65 - 74	39 (12.9%)
75 - 84	25 (8.3%)
≥85	2 (0.6%)
ASA Classifications	
Grade - I	112 (37%)
Grade - II	169 (55.8%)
Grade - III	22 (7.2%)
Grade - IV	0

Of the 303 patients included in the study, 178 (59%) were women and 125 (41%) were men. Comorbidities were assessed during the anaesthetic pre-assessment and comprehensively we had used ASA Grading to classify patients. Thirty-seven percent (37%) (112) of the patients included had an ASA score of I, 55.8% (169) had an ASA of II, 7.2% (22) had an ASA of III (Table 1).

Table 2 shows the distribution of the surgical procedure with respect to the surgical specialities and type of anaesthesia used. All the operations were classified by the operating team into ‘Minor’, ‘Intermediate’ and ‘Major’ complexity operations. Ninety-six percent (96%) (291) of the patients were discharged on the same day. Table 2 shows that only 4% (12) of the patients stayed more than 23 hours and their length of stay was mentioned further. Nine patients had an overnight stay for pain management and three patients stayed overnight for abdominal symptoms, which settled subsequently.

**Table 2:** Peri-operative variables of the study cohort. ENT: Ear, nose and throat

Total Patients (N)	303
Surgical Speciality	Cases Operated
Orthopaedics	66 (21.8%)
ENT	36 (11.8%)
General Surgery	62 (20.5%)
Gynaecology	60 (19.8%)
Plastic Surgery	13 (4.3%)
Urology	66 (21.8%)
Surgical complexity	
Minor	30 (9.9%)
Intermediate	171 (56.4%)
Major	102 (33.7%)
Type of Anaesthesia	
General	241 (79.6%)
Local & Regional	51 (16.8%)
Spinal	11 (3.6%)
Length of Stay	
<23 hrs	291 (96%)
>23 hrs	12 (4%)
Patient staying overnight	
24 - <48 hrs	9 (3%)
48 - <72 hrs	2 (0.7%)
>72 hrs	1 (0.3%)
Pre-operative COVID-19 Swab	
Done	303 (100%)
Pre-operative Swab Result	
Positive	0

On post-operative follow-up, there were no recorded incidence of pulmonary complications, the requirement of mechanical ventilation or death within 30 days of the index surgery (Table 3). No patients were readmitted after discharge. Only one patient who had COVID-19 suggestive symptoms in the post-operative period, was subsequently tested with an RT-PCR swab test with a negative result. No other patients contacted the hospital or their General Practitioner with any symptoms suggestive of COVID-19 infection.

**Table 3:** Post-operative variables for the study cohort. ARDS - Adult Respiratory Distress Syndrome

Hospital Readmissions	
Nil	0
Critical Care Admissions	
Nil	0
Pulmonary Complications	
No	303 (100%)
Pneumonia	0
ARDS	0
Mechanical Ventilation	0
NIV	0
30-Day Mortality	
Nil	0
Post-Operative Symptoms	1 (0.3%)
Post-Operative COVID-19 Swab	
Positive	0

Patients in this study received a telephone call to double-check for any suggestive symptoms after two weeks of their surgical procedure. A total of 86.1% (261 of 303) of the patients responded to the telephone follow-up questionnaire. However, we have gone through the electronic medical care record of all the patients. We have also tracked all the patients till 30 days from the surgery. We are not aware of any COVID-19 positive cases post-operatively.

## Discussion

The study reports the management and overall outcome of a series of 303 consecutive patients who underwent elective surgical procedures combining different specialities during the COVID‐19 pandemic. The outcomes show that the peri‐operative management plan for elective surgical procedures which we accomplished and delivered during this crucial time was safe and productive.

Because of the rapidly evolving pattern of the pandemic, the intra-operative and post-operative risks and outcomes for planned surgical procedures amidst the COVID-19 pandemic are partly understood. Few early studies reported that morbidity and mortality for surgical patients with added COVID‐19 infection might be significantly high. At the very onset of the pandemic, Lei et al. reported in a series of 34 patients from Wuhan who underwent elective surgery and were subsequently found to be COVID‐19 positive, 15 (44%) were admitted to intensive care and seven (20%) died [22]. In that study, patients were not screened for COVID‐19 infection pre‐operatively, only being tested once they became symptomatic [22]. An international multicentre observational study by COVIDSurg Collaborative reported 30‐day mortality of 23.8% on a combined cohort of 1128 emergency and elective surgical patients with peri‐operative infection with COVID‐19. They found 51.2% of patients suffering from pulmonary complications (adult respiratory distress syndrome; pneumonia; or unexpected postoperative ventilation) during the post-operative period [23]. While the mortality was greater following emergency surgery compared to elective procedures (26.0% vs. 19.1%, respectively), the likelihood of acquiring respiratory complications could be comparable for both groups [23]. Importantly, pulmonary complications accounted for 82.6% of all deaths. However, COVIDSurg Collaborative did not mention the number of patients with COVID‐19 negative results in the post-operative period within their study population. If this number is significantly high, the anticipated morbidity and mortality upon the background of peri‐operative risk might be low.

Kane et al. [24] from Middlesbrough, England reported seven out of 512 (1.4%) patients who underwent urgent elective surgery were identified as becoming COVID‐19 positive postoperatively, with one death. This one death of seven COVID‐19 positive patients (14.3%) is consistent with 20.4% mortality seen in elective patients in COVIDSurg Collaborative results. However, when this mortality is viewed within the cohort undergoing surgery (1/512 undergoing surgery), the added mortality risk due to COVID-19 is very low at 0.2%.

Gammeri et al. [25] reported a study of 309 consecutive elective surgical procedures with no major pulmonary complications and no 30-day mortality with the absence of important adverse outcomes. Their study cohort has significant demographic variability considering age group, gender and ASA classification of the patients and matches closely with our study cohort. Among the patients included in that study, 31.7% (98) had an ASA Grade of I, 58.6% (181) had an ASA of II, 9.4% (29) had an ASA of III compared to our study cohort of 37% (112) ASA Grade of I, 55.8% (169) had an ASA of II, 7.2% (22) had an ASA of III.

On the other hand, considering pre-operative RT-PCR testing, Kader et al. [26] have shown that the theoretical risk of a patient with an undetected COVID-19 infection being admitted for surgery and subsequently dying from the COVID-19 infection is estimated at approximately one in 7,000. Therefore, even if we consider the bias of a false-negative COVID-19 test (0.07%), the overall fatality following the infection could be low and it can be further lowered considering other strict peri-operative protocols.

It is definitely a matter of concern for nosocomial transmission of the COVID-19 infection, particularly, when we are bringing patients for elective or non-acute procedures. Columbia University Irving Medical Centre of the New York-Presbyterian Hospital have conducted a study to find out the incidence of nosocomial COVID-19 infection from March 1 till April 27, 2020, in two separate patient-care units restricted to COVID-19-negative patients (a cardiothoracic ICU and a regular floor unit). Hospital-acquired nosocomial transmission and infection with COVD-19 occurred in 0 to 2% of 311 patients [27,28]. These health care units studied were close to the wards adjacent, above, and below with COVID-19 positive patients. The study period also included the worst of the COVID-19 surge and plateau in New York City, and mitigation of infection was severely compromised, at least in March, by an additional scarcity of PPE. In spite of all these factors, the nosocomial transmission risk was found to be significantly low. However, not a universal standard, this can indirectly provide evidence for reinstating elective surgeries in a safe environment.

Cárdenas-Camarena et al. [29] published a meta-analysis reviewing 60 articles to address pre-operative protocols for patient selection for elective surgical procedures to enhance the safety threshold. They found that the combination of immunoglobulin M and immunoglobulin G antibody tests with RT-PCR (real-time polymerase chain reaction for COVID-19) conducted in different time frames by considering the natural course of the pandemic, it could be possible to reduce the risk of operating a patient during the incubation period by higher than 93%. Considering other peri-operative protective protocols can further increase this safety range. Therefore, we can precisely select the surgical candidates to curtail the incidence of peri-operative complications significantly.

Karayiannis et al. [30] published a study showing 30-day mortality of 1.9% among the patients undergoing orthopaedic trauma surgery in Northern Ireland during the peak of the COVID-19 pandemic. Although this study was conducted for non-elective surgeries in a COVID-19 hot site, they found no mortality for ASA Grade I and II patients. Therefore, reviewing their results amidst the most adverse situation, the result of this study can underpin the recent suggestion of resuming elective procedures relatively on low-risk patients. However, to be on the safer side, it would be good to start with low to minimal risk surgeries.

It is a known fact that COVID-19 infection and related complications are going to be a continuing risk factor for the upcoming future. On the other hand, the resumption of elective surgeries is almost a unanimous perception for all the nations which have passed the crucial waves of the pandemic. There could be several factors to be addressed when considering the reinstatement of elective services. Every patient needs to be individualised while considering surgical pre-assessment. Adequate consideration must be given to the factors like age, associated co-morbid conditions, potential for ongoing and previous exposure to the virus infection and any factor that might be contributing to an immunocompromised state. The threshold for risk stratification and anticipation of potential complications needs to be realistic while planning the elective surgery.

The result of this study is providing evidence for reinstating elective surgical procedures with a balance of risk stratification and anticipated complications. However, there is a need for a safe environment and robust peri-operative protocol which ensures minimum chance for nosocomial transmission. The locally implemented peri-operative management protocols for elective surgeries, that we followed during the pandemic with compliance to the Royal College and NHS guidelines, have been proven to be safe and can be considered as a framework for the resumption of elective services in near future.

We acknowledge the weakness of this single centre study with a moderate sample size. However, the characteristics of the study cohort and the outcomes are keeping in line with the available resources and literature at the time of writing. Certainly, there is a need for more evidence before establishing universal standards for elective procedures soon.

## Conclusions

Careful patient selection, the simultaneous involvement of the pre-assessment team and anaesthetic teams and strict adherence to the peri-operative protocols can help provide elective surgical services safely if the community transmission of the virus is under reasonable control. Moreover, delivering vigilant post-operative care for COVID-19 infection is crucial for maintaining the chain of safety. However, it is particularly important to maintain a COVID-free safe environment for such elective procedures.
